# WHICH NEIGHBORHOOD FEATURES MATTER MOST FOR MUSCLE STRENGTH? FINDINGS FROM THE HEALTH AND RETIREMENT STUDY

**DOI:** 10.1093/geroni/igac059.1010

**Published:** 2022-12-20

**Authors:** Kate Duchowny, L Grisell Diaz-Ramirez, W John Boscardin, Peggy Cawthon, Maria Glymour, Scarlett Lin Gomez

**Affiliations:** University of California, San Francisco, San Francisco, California, United States; University of California, San Francisco, San Francisco, California, United States; University of California, San Francisco, San Francisco, California, United States; California Pacific Medical Center Research Institute, San Francisco, California, United States; University of California, San Francisco, San Francisco, California, United States; University of California, San Francisco, San Francisco, California, United States

## Abstract

Linking data from the National Neighborhood Data Archive (NaNDA) to the 2006-2018 Health and Retirement Study (N=22,245), we fit linear mixed models to assess which of 22 built and social neighborhood environment variables predicted grip strength, a measure of total-body muscle strength. Among 22,245 respondents (mean age=63 years, SD=9.2) with up to 4 grip strength measures, neighborhood physical disorder (B= -0.25 kg, 95% CI= -0.37,-0.13), number of parks (B= 0.05 kg, 95% CI= 0.01, 0.10), number of gyms/fitness centers (B=-0.44 kg, 95% CI= -0.82, -0.07), proportion of highly developed land (B=-2.06 kg, 95% CI=-4.06, -0.07), and % urban (B=-0.66 kg, 95% CI=-1.27, -0.05) were associated with grip strength level after adjustment. No social neighborhood variables were associated with grip strength. Although preliminary, findings suggest that highly developed urbanized land may be a barrier to maintaining muscle strength in later life, but resources such as parks are associated with better outcomes.

